# Recurrent Aseptic (Mollaret) Meningitis: A Case Report

**DOI:** 10.7759/cureus.72137

**Published:** 2024-10-22

**Authors:** Lauren B Querin, Wayne A Martini, Byron S Parker

**Affiliations:** 1 Emergency Medicine, Mayo Clinic Arizona, Phoenix, USA; 2 Emergency Medicine, Veterans Affairs Medical Center, Prescott, USA

**Keywords:** aseptic meningitis, csf pleocytosis, herpes simplex virus type 2, mollaret meningitis, recurrent headache

## Abstract

Mollaret meningitis is a rare form of recurrent, self-resolving, aseptic meningitis with a high rate of return. Although it has been associated with several conditions, herpes simplex virus type 2 (HSV-2) is most common. Diagnosis requires recurrent (>3) episodes of aseptic meningitis as defined by pleocytosis in cerebrospinal fluid (CSF) with negative bacterial cultures. Management is controversial but focuses primarily on supportive care with a potential role for antiviral therapy in both treatment and prevention of recurrence. This case describes a 35-year-old male with fourteen prior episodes of meningitis presenting with a recurrence of symptoms. CSF studies showed an aseptic meningitis due to HSV-2. He was treated with antiviral therapy and ultimately discharged on daily valacyclovir for recurrence prevention. Increased awareness of this condition amongst emergency physicians may help reduce resource utilization and unnecessary antibiotics, while also enhancing appropriate follow-up and prevention of recurrence.

## Introduction

Headache is one of the most common symptoms leading to emergency department (ED) visits worldwide, and although most presentations are due to primary causes, it is essential that ED physicians consider secondary etiologies [1,2]. Meningitis is a secondary cause that should be considered; however, this diagnosis is rare, representing less than 1% of headache cases presenting to the ED [3]. Aseptic meningitis, inflammation of the meninges with negative bacterial cultures, represents >50% of meningitis cases in the US [4,5]. While most are singular occurrences, there are rare conditions, in which recurrent aseptic meningitis is seen. We describe the case of a young male who presented to an urban ED and was diagnosed with Mollaret meningitis, a rare form of recurrent aseptic meningitis.

## Case presentation

A 35-year-old male presented with one day of fever, headache, neck pain, and stiffness. There were no alleviating factors, however, the headache was worsened with movement and flexion of the neck. He had episodes of near identical symptoms in the past with a reported fourteen prior episodes of diagnosed meningitis. Past medical history was also notable for human immunodeficiency virus (HIV) and migraine headaches.

Initial vital signs included a blood pressure of 144/98 mm Hg, heart rate of 104 bpm, temperature 37.7ºC, and pulse oximetry of 97% on room air. On exam, he was uncomfortable, but alert and oriented. He had normal cardiopulmonary and abdominal exams, and no identifiable rashes or lesions. He had nuchal rigidity with positive Kernig and Brudzinski signs, but otherwise a non-focal neurologic exam.

In the ED, laboratory studies and a lumbar puncture were performed with cerebrospinal fluid (CSF) cell count illustrated in Table 1.

**Table 1:** CSF analysis consistent with aseptic meningitis. Cell count shows a pleocytosis with lymphocyte predominance, normal glucose, and elevated protein CSF: cerebrospinal fluid

Cell Count		Normal reference
Nucleated Cells/uL	44	<5
Neutrophils	19%	-
Lymphocytes	71%	-
Eosinophils	4%	-
Monocytes	6%	-
Glucose (mg/dL)	55	45-80
Protein (mg/dL)	68	23-38

CSF cell count showed a pleocytosis with lymphocyte predominance, normal glucose, and elevated protein, consistent with aseptic meningitis. Herpes simplex virus type 2 (HSV-2) polymerase chain reaction (PCR) of the patient’s CSF was positive, while HSV-1, COVID-19, and influenza A/B were negative. CSF gram stain had many white blood cells, but no organisms present. Given the patient's history of HIV, an absolute CD4 count was obtained and was 447 cells/μL (reference normal: 500-1500 cells/μL) suggesting a slightly immunocompromised status. He was given empiric intravenous 2 g ceftriaxone, 2,000 mg vancomycin, and 700 mg acyclovir in addition to symptomatic treatment. Acyclovir was continued during the inpatient stay, while antibiotics were discontinued after the results of the CSF studies were available. The patient was ultimately discharged home on hospital day three with significant symptomatic improvement and the diagnosis of Mollaret meningitis. Discharge medications included 1 g of oral valacyclovir twice daily indefinitely for prevention of recurrence. 

## Discussion

Mollaret meningitis is a rare form of recurrent, self-resolving, aseptic meningitis that can significantly impact patient quality of life [6,7]. As seen in this case, the major underlying etiology associated with Mollaret meningitis is HSV-2, isolated in up to 85% of cases [8]. Other associated conditions include HSV-1, varicella-zoster virus, HIV, enterovirus, intracranial cysts or tumors, sarcoidosis, and systemic lupus erythematosus [9]. Although Mollaret meningitis has been associated with immunodeficiency, such as HIV as seen in this patient's case, there are also previously reported cases in immunocompetent individuals [6,9].

Clinical presentation mimics that of other underlying causes of meningitis, including headache, fever, neck stiffness, and positive meningeal signs. Although transient neurologic deficits can be seen, Mollaret meningitis is not typically associated with long-term neurologic sequelae [9]. Symptoms persist for several days before self-resolution and there are typically symptom-free intervals in between episodes [6,9]. Episode frequency is variable but may occur as often as every few weeks [7]. Fortunately, long-term complications are rare; however, there have been reports of post-meningitis migraines, as seen in this case [9]. 

ED evaluation should align with standard meningitis work-up, which includes serum laboratory analysis, blood cultures, and a lumbar puncture for CSF. Head imaging is typically not necessary, and physicians should follow the same practice patterns they would use for patients being evaluated for meningitis [2]. CSF should be sent for cell count, culture, glucose, and protein. A viral PCR, including testing for HSV, should be performed [8,10]. 

Diagnosis of Mollaret meningitis requires three recurrent episodes with spontaneous resolution of signs and symptoms, followed by symptom-free periods between episodes [9,11]. CSF studies should demonstrate pleocytosis with elevated protein, and normal glucose. Although HSV-2 is commonly detected, an underlying viral source is not required for diagnosis. Very early in the course of the disease, large endothelial cells, often referred to as Mollaret cells, may be seen. They are rarely detected; however, they are highly fragile and typically disappear within 24 hours of symptom onset [9,12].

Treatment is centered around symptomatic management. Empiric antivirals and antibiotics are often administered, especially in the setting of diagnostic uncertainty or poor rapidity of CSF analysis. Patients with suspected Mollaret meningitis are commonly admitted to the hospital; however, definitive treatment remains controversial [9,12,13]. Most patients experience spontaneous symptom resolution after several days and the benefit of antiviral medication remains debatable. While some reports have demonstrated improvement with chronic valacyclovir suppressive therapy, others suggest that there is no role for antivirals in either the treatment or prevention of symptoms [6,10,14,15]. Most patients receive consultation with infectious disease or neurology for decisions regarding prophylactic antiviral medication and alternative strategies for recurrence prevention.

## Conclusions

Mollaret meningitis is a rare form of recurrent aseptic meningitis that can be debilitating for patients and result in prolonged hospital admissions. This article highlights a case of Mollaret meningitis and reviews the literature regarding diagnostic criteria and management of this condition, including the role of chronic suppressive therapies. Increased awareness of this condition among emergency physicians, inpatient hospitalists, and neurologists may help reduce resource utilization while also enhancing appropriate follow-up and prevention of recurrence.
